# Factors associated with post-operative delirium in hip fracture patients: what should we care

**DOI:** 10.1186/s40001-022-00660-9

**Published:** 2022-03-12

**Authors:** Dequn Kong, Weihua Luo, Zhijun Zhu, Sixin Sun, Jian Zhu

**Affiliations:** Department of Orthopedics, Taixing People’s Hospital, No. 1 Changzheng Road, Taixing, 225400 Jiangsu China

**Keywords:** Delirium, Surgery, Hip fracture, Treatment, Care, Prevention

## Abstract

**Background:**

The postoperative delirium is a common yet serious complication in elderly patients with hip fracture. We aimed to evaluate the potential risk factors of delirium in patients with hip fracture, to provide reliable evidence to the clinical management of hip fracture.

**Methods:**

This study was a retrospective design. Elderly patients who underwent hip fracture surgery in our hospital from June 1, 2019 to December 30, 2020 were selected. The characteristics and treatment data of delirium and no delirium patients were collected and compared. Multivariate logistic regression analysis was performed to analyze the influencing factors affecting postoperative delirium in elderly patients with hip fracture.

**Results:**

A total of 245 patients with hip fracture were included, the incidence of postoperative delirium in patients with hip fracture was 13.06%. There were significant differences in the age, BMI, history of delirium, estimated blood loss and duration of surgery (all *p*  < 0.05). There were significant differences in the albumin and TSH between delirium and no delirium group (all *p* < 0.05), Logistics analyses indicated that age ≥ 75 years (OR 3.112, 95% CI 1.527–5.742), BMI ≥ 24 kg/m^2^ (OR 2.127, 95% CI 1.144–3.598), history of delirium (OR 1.754, 95% CI 1.173–2.347), estimated blood loss ≥ 400 mL (OR 1.698, 95% CI 1.427–1.946), duration of surgery ≥ 120 min (OR 2.138, 95% CI 1.126–3.085), preoperative albumin ≤ 40 g/L (OR 1.845, 95% CI 1.102–2.835) and TSH ≤ 2 mU/L (OR 2.226, 95% CI 1.329–4.011) were the independent risk factors of postoperative delirium in patients with hip fracture(all *p* < 0.05).

**Conclusions:**

Postoperative delirium is very common in elderly patients with hip fracture, and it is associated with many risk factors, clinical preventions targeted on those risk factors are needed to reduce the postoperative delirium.

**Supplementary Information:**

The online version contains supplementary material available at 10.1186/s40001-022-00660-9.

## Background

With the aging of the population, the incidence of hip fractures is increasing year by year [1]. It is reported that the incidence of hip fractures is 11.1% in men and 22.7% in women [2, 3]. Almost half of hip fractures occur in patients 70 years of age or older. It is estimated that 18–28% of elderly hip fracture patients die within a year [4]. Hip fractures in the elderly seriously affect their quality of life and bring about a lasting decline in physical fitness, which leads to an increase in the morbidity and mortality [5]. Postoperative delirium is the most common type of complication after hip fracture [6, 7]. Delirium is a kind of acute mental disorder with manifestation of decrease in concentration, sustained ability, and easy to be interfered with [8]. During the illness, there are fluctuations in the condition every day, usually manifested as daytime sleepiness and night irritability. Studies [9, 10] have reported that patients with delirium hip fractures during hospitalization have a worse prognosis than those without delirium. Delirium during the perioperative period will seriously affect the patient’s treatment effect, increase the mortality or the need for home care after discharge, length of hospital stays, costs, and hospital-acquired complications [11, 12].

In clinical practice, due to the lack of knowledge of medical staff and the complexity and change of symptoms, about 32. 46% of delirium is often undetected or not treated, which will bring more serious consequences to the patient [13]. Thirty to Forty percent of perioperative delirium can be prevented, and effective prevention of delirium can minimize its occurrence and related adverse consequences [14, 15]. Therefore, we aimed to analyze the characteristics and related influencing factors of postoperative delirium in patients with hip fractures, to provide evidence support for the prevention of postoperative delirium and management of clinical hip fractures.

## Methods

### Ethics

In this study, all methods were performed in accordance with the relevant guidelines and regulations. This present study was a retrospective design. Our study had been verified and approved by the ethical committee of our hospital (Approval Number: LS2019015). Our hospital is an urban tertiary hospital with designated 1788 beds for medical services. For patients with delirium, the written informed consent from guardians for such patients were obtained. For the patients without delirium, written informed consents were obtained from patients.

### Patients

Elderly patients who underwent hip fracture surgery in the orthopedics department of our hospital from June 1, 2019 to December 30, 2020 were selected as the research patients. The inclusion criteria for patients in this study were: age ≥ 60 years; the hip fracture met the diagnostic criteria for hip fractures; patient underwent elective surgery in our hospital; all patients and their families had been informed and signed an informed consent form; clinical data was complete. Exclusion criteria: patients with a history of brain surgery; participants experienced delirium before the surgical procedure; patients with hearing impairment, since we might have difficulty to communicate with; patients with malignant tumors, since they were mainly treated in the oncology department of our hospital; patients taking sedatives or antidepressants; patients who were unwilling to participate in this study.

### Diagnosis of delirium

According to the diagnostic criteria in the fifth edition of the Diagnostic and Statistical Manual of Mental Disorders (DSM-V) of the American Psychiatric Association [16], the confusion assessment method (CAM) was used as a diagnostic tool for the diagnosis of delirium [17]. CAM includes 4 items: ① acute onset and fluctuating changes in mental state; ② inattention; ③ thinking disorder; ④ change of consciousness level. The CAM assessment was conducted for all the patients pre-operation and post-operation twice a day until the discharge of patients. All the nurses and doctors in our department had been trained for the CAM assessment. The CAM assessment was conducted and recorded by the nurse in charge.

### Data collection

Two authors independently collected following clinical data: age, gender, body mass index (BMI), hypertension, diabetes mellitus, hyperlipidemia, history of delirium, type of fracture, time to surgery, postoperative ability of daily living (ADL) score, method of anesthesia, estimated blood loss and duration of surgery. Besides, all patients underwent preoperative laboratory examinations. We collected following biochemical indicators: albumin, hemoglobin, blood sugar, creatinine, urea nitrogen, triiodothyronine (T3), thyroxine (T4), serum thyroid stimulating hormone (TSH), free triiodothyronine (FT3), free thyroxine (FT4), arterial partial pressure of oxygen (PaO_2_) arterial partial pressure of carbon dioxide (PaCO_2_).

### Statistical method

We used SPSS 25. 0 Software to carry out statistical analysis of relevant data. Measurement data was expressed as mean ± standard deviation, measurement data between groups was compared by independent sample *t* test, count data was expressed by number of cases and percentage, and count data were compared by Chi-square test. The covariates with statistical significance in the univariate analysis were further included into the multivariate logistic regression model. We used multivariate logistic regression to analyze the factors affecting postoperative delirium in elderly patients with hip fracture. In this study, *p* < 0. 05 meant that the difference between the groups was statistically significant.

## Results

### The characteristics of included patients

A total of 245 patients with hip fracture were included in this study, and 32 patients had been diagnosed as delirium, the incidence of postoperative delirium in patients with hip fracture was 13.06%. As shown in Table 1, there were significant differences in the age, BMI, history of delirium, estimated blood loss and duration of surgery (all *p* < 0.05). No significant differences in the gender, hypertension, diabetes mellitus, hyperlipidemia, type of fracture, time to surgery, postoperative ADL score and method of anesthesia were found (all *p* > 0.05).

**Table 1:** Characteristics of included patients

Items	Delirium group (*n* = 32)	No delirium group (*n* = 213)	*t*/*χ*^2^	*p*
Age (years)	78.84 ± 7.36	71.21 ± 5.83	10.731	0.014
Male/female	12/20	84/129	1.926	0.085
BMI (kg/m^2^)	25.32 ± 3.56	21.20 ± 2.98	1.142	0.026
Hypertension	21 (62.63%)	110 (51.64%)	1.165	0.101
Diabetes mellitus	13 (40.63%)	78 (36.62%)	1.224	0.094
Hyperlipidemia	14 (43.75%)	82 (38.50%)	1.707	0.089
History of delirium	17 (5 3.13%)	19 (8.92%)	1.113	0.021
Type of fracture			1.188	0.107
Femoral neck fracture	13 (40.63%)	81 (38.03%)		
Intertrochanteric fracture	18 (56.25%)	129 (60.56%)		
Subtrochanteric fracture	1 (3.12%)	3 (1.41%)		
Preoperative ADL score	95.42 ± 7.18	94.95 ± 9.84	22.107	0.073
Time to surgery (days)	1.84 ± 1.04	1.77 ± 1.16	1.842	0.109
Method of anesthesia			1.882	0.096
Lumbar anaesthesia	19 (59.38%)	124 (58.22%)		
General anaesthesia	13 (40.63%)	89 (41.78%)		
Estimated blood loss (mL)	455.07 ± 62.85	369.12 ± 58.23	38.467	0.002
Duration of surgery (min)	147.12 ± 36.77	102.37 ± 24.16	19.236	0.008

### Preoperative laboratory examination results

As shown in Table 2, there were significant differences in the albumin and TSH between delirium and no delirium group(all *p* < 0.05), no significant differences in the hemoglobin, blood sugar, creatinine, urea nitrogen, T3, T4, FT3, FT4, PaO_2_ and PaCO_2_ were found(all *p* > 0.05).

**Table 2:** Preoperative laboratory examination results

Items	Delirium group (*n* = 32)	No delirium group (*n* = 213)	*t*/*χ*^2^	*p*
Albumin (g/L)	38.05 ± 6.22	44.96 ± 5.34	7.116	0.025
Hemoglobin (g/L)	122.03 ± 19.74	120.84 ± 14.09	17.253	0.091
Blood sugar (mmol/L)	7.42 ± 1.08	7.51 ± 1.33	1.650	0.102
Creatinine (μmol/L)	76.95 ± 8.31	75.32 ± 8.84	6.041	0.087
Urea nitrogen (mmol/L)	7.73 ± 1.02	7.24 ± 1.27	1.636	0.099
T3 (mU/L)	1.41 ± 0.12	1.38 ± 0.11	1.204	0.112
T4 (mU/L)	95.03 ± 12.54	96.23 ± 14.04	8.345	0.063
TSH (mU/L)	1.62 ± 0.22	2.74 ± 0.52	1.224	0.022
FT3 (mU/L)	3.13 ± 0.97	3.30 ± 0.83	1.137	0.059
FT4 (mU/L)	16.84 ± 4.03	16.59 ± 5.18	3.284	0.064
PaO_2_ (mmHg)	84.36 ± 12.65	86.89 ± 15.11	9.705	0.131
PaCO_2_ (mmHg)	33.82 ± 5.78	32.41 ± 6.05	4.822	0.061

### Logistic regression analyses

The variable assignment of multivariate logistic regression was presented in Additional file 1. As indicated in Table 3, Age ≥ 75 years (OR 3.112, 95% CI 1.527–5.742), BMI ≥ 24 kg/m^2^ (OR 2.127, 95% CI 1.144–3.598), history of delirium (OR 1.754, 95% CI 1.173–2.347), estimated blood loss ≥ 400 mL (OR 1.698, 95% CI 1.427–1.946), duration of surgery ≥ 120 min (OR 2.138, 95% CI 1.126–3.085), preoperative albumin ≤ 40 g/L (OR 1.845, 95% CI 1.102–2.835) and TSH ≤ 2 mU/L (OR 2.226, 95% CI 1.329–4.011) were the independent risk factors of postoperative delirium in patients with hip fracture(all *p* < 0.05).

**Table 3:** Logistic regression analysis on the risk factors of postoperative delirium in patients with hip fracture

Items	*β*	SE	OR	95% CI	*p*
Age ≥ 75 years	0.122	0.495	3.112	1.527–5.742	0.012
BMI ≥ 24 kg/m^2^	0.217	0.356	2.127	1.144–3.598	0.029
History of delirium	0.143	0.518	1.754	1.173–2.347	0.042
Estimated blood loss ≥ 400 mL	0.257	0.522	1.698	1.427–1.946	0.013
Duration of surgery ≥ 120 min	0.187	0.236	2.138	1.126–3.085	0.046
Albumin ≤ 40 g/L	0.124	0.331	1.845	1.102–2.835	0.017
TSH ≤ 2 mU/L	0.114	0.642	2.226	1.329–4.011	0.009

## Discussion

Postoperative delirium is an acute psychopathic syndrome with cognitive dysfunction and other related symptoms including disturbances in consciousness, perception, thinking, mood, memory, attention, and sleep cycle [18]. Postoperative delirium has a high morbidity and related mortality in elderly patients [19]. According to reports [20, 21], the incidence of delirium after hip fracture in the elderly ranges from 5.15 to 48.37%, which may be related to the differences of diagnostic criteria of delirium. Previous study [22] has analyzed 232 cases of elderly patients with hip fracture and has found that delirium occurred in 30.2% of patients, and the survival rate of this part of patients 40 months after surgery was 63.6%, which was much lower than the normal patient’s survival rate of 81.0%. Besides, previous studies [23–25] have found that patients with delirium have longer hospital stays, higher medical costs, and the incidence of dementia after discharge can be as high as 38%, and postoperative hip function and independent living ability of delirium patients are difficult to restore to the preoperative level, which seriously affects the prognosis of patients. Therefore, early prevention, detection and treatment of delirium have important clinical significance for improving the prognosis of patients with hip fracture. The results of our study have indicated that the incidence of postoperative delirium in patients with hip fracture is 13.06%, and age ≥ 75 years, BMI ≥ 24 kg/m^2^, history of delirium, estimated blood loss ≥ 400 mL, duration of surgery ≥ 120 min, preoperative albumin ≤ 40 g/L and TSH ≤ 2 mU/L are the independent risk factors of postoperative delirium in patients with hip fracture. Clinical measures targeted on those risk factors are needed to prevent the onset and development of postoperative delirium.

With the increase of age, the organs and tissues of elderly patients degenerate and their functions decline, and there are often many chronic underlying medical diseases, such as the cardiovascular system, respiratory system, and endocrine system [26]. Besides, they are also easy to merge due to decreased appetite and intake, and metabolic disorders, leading to malnutrition [27]. Studies [28, 29] have shown that elderly patients with hip fractures ≥ 73 years of age are 1.83 times more likely to develop delirium after surgery than those aged < 73 years. Studies [30, 31] have shown that age is attributable to preoperative malnutrition in elderly patients with medullary fractures, and it is one of the main risk factors of delirium in elderly patients, which is consistent with the results of this study.

Higher BMI is associated with higher risk of delirium. It’s been reported that obesity is associated with the development of delirium, and obesity patients generally have higher BMI [32, 33]. Besides, it’s been reported that the higher BMI is associated with hip fracture in elderly patients [34]. The association of BMI and higher risk of delirium may be a J-shaped curve, since the obesity is generally defined as BMI ≥ 28 kg/m^2^. Limited by sample size, we could not perform subgroup analysis on the association of BMI and delirium, future studies with larger sample size and different population are needed.

The duration of surgery is a risk factor for delirium after hip fracture surgery in the elderly. The reasons may be that the prolonged operation duration means prolonged anesthesia time and stress duration of surgical trauma [35]. However, the function of various organs in elderly hip patients has a certain degree of degradation, and then suffers a longer surgical trauma and stress level, the body is more likely to be decompensated, and the central system of norepinephrine–acetylcholine is more likely to be unbalanced, causing brain to be extremely excited, leading to the occurrence of delirium [36, 37]. In addition, the prolonged surgery duration for elderly patients with hip fractures also means increased surgical trauma and intraoperative blood loss, excessive blood loss has aggravated the hypoxia of brain cells, resulting in the occurrence of delirium [38]. It’s been reported that the intraoperative anesthetics is associated with the delirium, yet we did no find this association, which may be explained that the sample size is not large enough to detect the association. Therefore, preoperative doctors should formulate individualized surgical plans according to the specific conditions of the patients, speed up the operation process to reduce intraoperative blood loss, shorten the operation time, and reduce the use of intraoperative anesthetics.

Low albumin is one of the manifestations of patients with malnutrition. It’s been reported that the patient’s nutritional status has a certain correlation with the occurrence of postoperative delirium [39]. Studies [40, 41] have shown that the risk of postoperative delirium among elderly patients with hip fracture is 3 times that of those with normal nutrition and 2.5 times that of those with malnutrition. There may be an underlying inflammatory process causing this. Thus, factors associated with prolonged inflammation, such as infections, delay in surgery may be involved. Some studies [42, 43] believe that the application of high-protein nutritional supplements before surgery can improve the clinical outcome of surgical patients. Therefore, the monitor and correction of albumin level is vital to the prognosis of patients with hip fracture surgery.

Hip fracture surgery is traumatic and usually takes a long time, thus the postoperative stress response is obvious. It disrupts the hypothalamus–pituitary–thyroid axis of the patient and affects thyroid function [44]. Thyroid hormone is an essential substance for maintaining the normal function of the central nervous system and is closely associated with human mental activity [45]. Previous studies [46, 47] have shown that the inflammatory response of the systemic system caused by postoperative stress can lead to delirium by increasing the permeability of the blood–brain barrier. Thyroid hormone can significantly affect the body’s protein metabolism, has a diuretic effect and stimulating effect on the function of the adrenal cortex, and affects the function of the cardiovascular system, thereby affecting the blood flow of important organs, such as the liver, kidney, and brain. As the number of brain cells in the elderly decreases year by year with age, the cerebral blood flow is also reduced, the metabolic level is significantly reduced, the central neurotransmitter is reduced, and the nerve conduction speed slows down, resulting in widespread brain dysfunction. Coupled with the primary disease and surgical trauma, the combined effect of other influencing factors makes elderly patients become prone to postoperative delirium [48, 49].

This study has certain shortcomings that must be considered. First, the sample size of the study included in this study is small, it maybe underpower to detect the group differences. In addition, we have excluded patients with hearing impairment and malignancy, it may be useful to evaluate the influences of those factors on delirium. Second, this study is a retrospective study design, the included indicators are limited, dementia or mild cognitive impairment are not considered as covariates in the study, considering the known association between dementia and delirium. Future prospective studies are needed to further analyze the related factors of postoperative delirium. Third, we have made the follow-up until the discharge of patients, we have not performed follow-up after hospital discharge, the long-term risk of delirium remains unclear. Finally, there is a certain difference between the incidence of delirium in this study and previous related studies. The reason may be due to the different diagnostic criteria and postoperative follow-up time for delirium, the possibility of internal bias in the study cannot be ruled out. Therefore, future studies with larger samples size are needed to further elucidate the influencing factors of postoperative delirium.

## Conclusions

In conclusion, we have found that postoperative delirium is very common is patients with hip fracture, and for patients with age ≥ 75 years, BMI ≥ 24 kg/m^2^, history of delirium, estimated blood loss ≥ 400 mL, duration of surgery ≥ 120 min, preoperative albumin ≤ 40 g/L and TSH ≤ 2 mU/L, they may have higher risks for the development of postoperative delirium, early alert and preventions should be taken in advance to reduce the onset of postoperative delirium. Still, limited by sample size and study design, future studies with larger sample and rigorous design are highlighted to further ascertain the risk factors of postoperative delirium in patients with hip fracture, to provide reliable evidence to the management of hip fracture.

## Supplementary Information

**Additional file 1: Table S1.** The variable assignment of multivariate logistic regression.

## Data Availability

All data generated or analyzed during this study are included in this published article.

## Abbreviations

DSM-V: Diagnostic and statistical manual of mental disorders
CAM: Confusion assessment method
BMI: Body mass index
ADL: Ability of daily living
T3: Triiodothyronine
T4: Thyroxine
TSH: Thyroid stimulating hormone
FT3: Free triiodothyronine
FT4: Free thyroxine
PaO_2_: Arterial partial pressure of oxygen
PaCO_2_: Arterial partial pressure of carbon dioxide
