# Supplementary material for: Factors associated with post-operative delirium in hip fracture patients: what should we care
Source: Eur J Med Res. 2022 Mar 12;27:40. doi: 10.1186/s40001-022-00660-9 (PMC8917680; doi:10.1186/s40001-022-00660-9)
Supplement: Supplementary file 1 — Additional file 1: Table S1. The variable assignment of multivariate logistic regression. [file 40001_2022_660_MOESM1_ESM.docx]

Table S1 The variable assignment of multivariate logistic regression

| Factors | Variables | Assignment |
| --- | --- | --- |
| Delirium | Y | Yes=1, no=2 |
| Age(y) | X_1_ | ≥75y=1, <75y=2 |
| BMI(kg/m^2^) | X_2_ | ≥24=1, <24=2 |
| History of delirium | X_3_ | Yes=1, no=2 |
| Estimated blood loss(ml) | X_4_ | ≥400=1, <400=2 |
| Duration of surgery(min) | X_5_ | ≥120=1, <120=2 |
| Albumin(g/L) | X_6_ | ≤40=1, >40=2 |
| TSH(mU/L) | X_7_ | ≤2=1, >2=2 |
